# Optimizing carrier collection in solar cells through nanoscale junction design

**DOI:** 10.1039/d5ya00251f

**Published:** 2026-03-19

**Authors:** Melanie Micali, Raphaël François Lemerle, Anja Tiede, Anna Fontcuberta i Morral, Esther Alarcón-Lladó

**Affiliations:** a NWO-I Institute AMOLF, Science Park 104 1098XG Amsterdam The Netherlands M.Micali@amolf.nl E.Alarconllado@amolf.nl; b Laboratory of Semiconductor Materials, Institute of Materials, Ecole Polytechnique Fédérale de Lausanne 1015 Lausanne Switzerland; c Institute of Physics, Ecole Polytechnique Fédérale de Lausanne 1015 Lausanne Switzerland; d Van ’t Hoff Institute for Molecular Sciences (HIMS), University of Amsterdam 1090GD Amsterdam The Netherlands

## Abstract

A key challenge in thin-film photovoltaics is achieving selective carrier collection that minimizes recombination losses while maintaining efficient charge extraction. This study presents a theoretical analysis of how reducing junction contact area can enhance the open-circuit voltage (*V*_OC_) and the power conversion efficiency (PCE) in thin-film solar cells. Using a zinc-phosphide (Zn_3_P_2_) -based heterojunction as a model, we simulate the effect of geometrically minimizing contact *via* silicon-dioxide (SiO_2_) layers with patterned holes. The smaller the contact area, the lower the reverse saturation current, which results in a significant increase in the *V*_OC_ up to 100 mV. However, the reduced contact area also increases the series resistance, thereby limiting the gain in PCE. This approach is especially effective with non-absorbing highly-doped transport layers, such as titanium-dioxide (TiO_2_) (PCE gain up to 1.45%). This work underscores the importance of balancing reduced recombination with parasitic resistance and current crowding for optimal performance.

## Abstract 2

This work shows how purely the junction geometry can improve the open circuit voltage in thin film solar cell devices.

## Graphical abstract

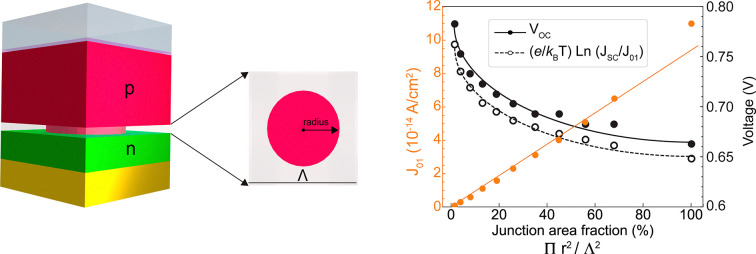

## 1 Introduction

The performance of thin-film solar cells is particularly limited by *V*_OC_ losses that directly impact the PCE of the device.^1,2^ The upper threshold for the *V*_OC_ is governed by the thermodynamic limit^3,4^ to below the semiconductor bandgap due to unavoidable radiative recombination losses. Additional losses, such as non-radiative recombination mechanisms (Shockley–Read-Hall, Auger or surface recombination), non-appropriate band-alignment, incomplete light absorption, or carrier transport losses, can significantly further reduce the *V*_OC_ below the radiative limit.^5^ Many efforts have been devoted to increasing *V*_OC_ by using layers at the contacts that minimize recombination losses. New technologies, including BSF,^6^ PERL,^7^ PESC,^8^ PERC,^9^ and TOPCon,^10^ have demonstrated that it is possible to reduce rear and front surface recombination through highly doped regions and SiO_2_ passivating layers, respectively. These solutions are closely tied to the intrinsic material properties and the techniques used in processing and fabrication. Decoupling and mitigating these voltage losses from the material's characteristics can be crucial for enhancing solar cell efficiencies, particularly for materials whose property control is challenging or costly.

An alternative strategy to minimize recombination losses is to reduce the contact area *via* the creation of local contacts. This is a purely geometric approach to enhance *V*_OC_, that takes advantage of the relation between *V*_OC_ and the reverse saturation current density (*J*_0_).^5^ In the 1980's it was already predicted that an array of dot-shaped contacts was the most promising geometry to maximize the voltage output in silicon solar cells.^11,12^ In this case, the metal contacts touch the silicon absorber in a patterned array of small dots, each aligned with a localized diffused region in the silicon. More recently, nanoscale confined junctions have been proposed for perovskite-based cells, where the confined contact geometry not only offers a reduced junction recombination but also allows for combining surface passivation and carrier collection in one layer,^13,14^ or the design of fully back-contacted devices.^15–17^

The solution processing and defect tolerance of perovskite materials facilitate the integration of nanoscale contacts. However, the adoption of nanoscale contacts in other thin-film solar cell technologies is less common, as multi-material interfaces with intricate nanoscale features make it challenging to preserve the film's crystalline quality. Selective area epitaxy (SAE) addresses this challenge by enabling controlled epitaxial growth only on exposed substrate regions through a patterned dielectric mask,^18^ with the option to extend growth laterally using lateral epitaxial overgrowth techniques.^18–24^ Recently, advances in SAE have even demonstrated precise control over the growth of crystalline films and nanostructures from earth-abundant absorber materials, including Zn_3_P_2_,^25–28^ offering new opportunities for integrating nanoscale contacts without compromising crystalline quality. The fabrication feasibility of controlled nanoscale areas enables the engineering of nanoscale contacts at the interfaces that preserve material quality and actively enhance photovoltaic performance. While these studies establish the feasibility of SAE for earth-abundant absorbers such as Zn_3_P_2_, a systematic investigation of its potential for the incorporation of nanoscale contacts in photovoltaic architectures is still lacking.

In this work, we present a theoretical analysis of how nanoscale contact geometry can be leveraged to improve the performance of thin-film solar cells. Using a Zn_3_P_2_ -based heterojunction as a model, we investigate the effect of reducing the junction contact area through patterned SiO_2_ layers with nanoscale openings. Zn_3_P_2_ possesses all the key material properties required for the development of thin-film solar cells. It is an emerging light absorber with a direct bandgap of 1.5 eV, close to the maximum theoretical efficiency predicted by the detailed balance limit. Moreover, Zn_3_P_2_ exhibits a high absorption coefficient (10^4^–10^5^ cm^−1^), promising carrier diffusion lengths (10 µm), and high carrier mobility.^29–31^ In addition, it is earth-abundant and easily recyclable. By coupling 3D optical and electrical simulations, we quantify how smaller contact areas lower the reverse saturation current and increase *V*_OC_ by up to 120 mV, while also assessing the trade-offs introduced by higher series resistance. We identify conditions where this strategy delivers a PCE gain of up to 1.45% (absolute), providing design guidelines for balancing recombination suppression with parasitic resistance and current crowding. This work addresses a gap in the literature by systematically evaluating the competing mechanisms that arise from reduced-area junctions and by outlining their practical implications for designing high-efficiency, earth-abundant photovoltaics.

## 2 Results and discussion

### 2.1 Impact of the junction area fraction on the device characteristics

**Fig. 1:**
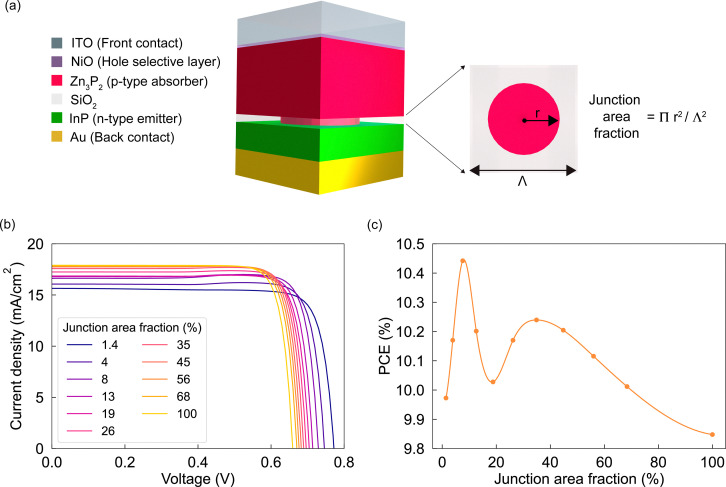
(a) Schematic representation of the device stack. Simulated (b) *JV* curves under 1 Sun illumination and (c) solar cell efficiency (PCE) as a function of the InP/Zn_3_P_2_ junction area fraction.

Fig. 1a illustrates the simulated device stack, which comprises a 250 nm-thick p-type Zn_3_P_2_ absorber layer, contacted at the front by a 10 nm-thick NiO hole-selective layer (HSL) and at the rear by a highly doped InP n-type emitter. To modulate the p–n contact area, an intermediate 30 nm-thick SiO_2_ insulating layer is introduced, incorporating Zn_3_P_2_ -filled cylindrical holes in a periodic square array. The junction area fraction (or contact area) is defined as:1
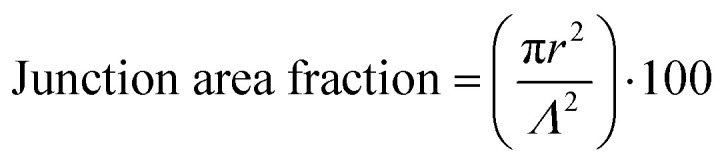
where the hole radius is varied in the range *r* = [30–210] nm and the pitch is fixed to *Λ* = 450 nm. The electrical contacts are set as ITO (front) and Au (back) for the optical simulations. For electrical simulations, the contacts are simply considered ohmic with flat band alignment with the adjacent layers. The main purpose of the study was to examine the purely geometric effects on the device performance. For this reason, several simplifying assumptions were made. In particular, the model does not account for interface traps at the interface between the SiO_2_ mask and the absorber layer. More details on the simulation setup and material parameters can be found in the Methods and SI sections, respectively.

Fig. 1b shows simulated *JV* curves under AM1.5G illumination for various junction area fractions. Two dominant effects emerge as the junction area fraction decreases: (1) the *V*_OC_ increases monotonically, and (2) the short-circuit current density *J*_SC_ decreases modestly (see also Fig. S1a and b). The resulting PCE and fill factor (FF), shown in Fig. 1c and Fig. S1c, respectively, exhibit a non-monotonic trend. This reflects a trade-off between voltage gains and current losses, leading to a maximum PCE of 10.44% at a junction area fraction of 8%. This corresponds to an absolute efficiency improvement of approximately 0.5% over the full-contact (100%) configuration.

### 2.2 The role of the saturation current and parasitic resistances

To investigate the origin of the observed changes in solar cell performance, we simulate the dark *JV* characteristics and analyze them using a two-diode equivalent circuit model^32^ (see Section SI.3). The model consists of two diodes in parallel (one with ideality factor *n* = 1 representing bulk recombination, and one with *n* = 2 representing depletion-region recombination) along with a shunt resistance connected in parallel and series resistance contributions. Each element contributes to the total current density *J*, with the shunt resistance primarily influencing device behavior under low or reverse bias.

**Fig. 2:**
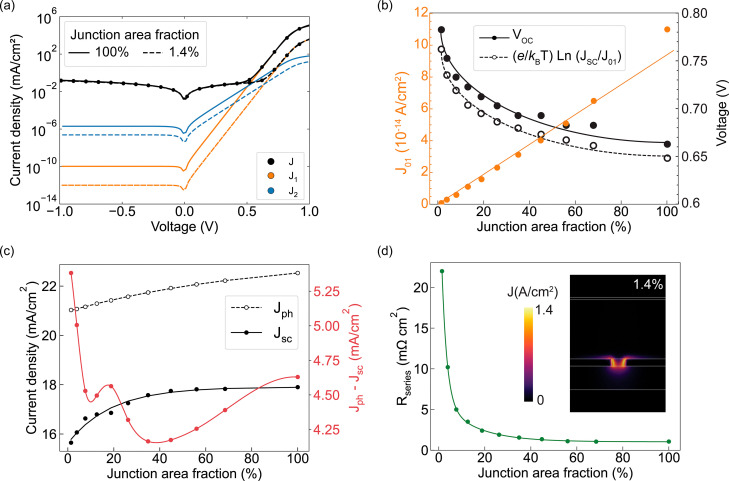
(a) Simulated dark *JV* characteristics (black dots) and 2-diode model fit (black curves) for the cases of full contact (solid curve) and nano-contact (dashed curve). The orange and blue curves represent the contributions to the current of the first and second diodes (*J*_1_ and *J*_2_), respectively. (b) Extracted dark current (*J*_01_, orange curve), simulated *V*_OC_ (filled black dots), and estimated *V*_OC_ (empty dots) as a function of the InP/Zn_3_P_2_ junction area fraction. The potential is estimated by assuming the expression in the legend. (c) *J*_ph_ (black-dashed curve), *J*_SC_ (black-solid curve) and relative difference *J*_ph_–*J*_SC_ (red-solid curve) as a function of the InP/Zn_3_P_2_ junction area fraction. (d) *R*_*series*_ extracted from the fit of the dark *JV* curves as a function of the junction area fraction. The inset is the vertical cross-section distribution of the current at *V* = 0, for a junction area fraction of 1.4%. White lines delimit the different layers of the cell.

Fig. 2a shows the dark *JV* curves in log-scale for the two extreme conditions: a full area contact (black solid curve) and the smallest nano-contact geometry (*i.e.* 1.4% of contact area fraction, black dotted curve). From the 2-diode model fit, we extract the components associated with diffusion-governed current (*J*_1_, orange curves) and interface recombination current (*J*_2_, blue curves). While the saturation current density *J*_01_ is four orders of magnitude smaller than *J*_02_ (identified in the reverse bias region), the exponential increase in its current at high forward bias causes diode 1 to dominate the total current, making it the primary factor that sets *V*_OC_. The breakdown of *J*_1_ and *J*_2_ in Fig. 2a confirms that the majority of the recombination at high voltage occurs through *J*_1_, and that its reduction *via* this geometrical strategy drives the *V*_OC_ enhancement. This is further confirmed in Fig. 2b, where the simulated *V*_OC_ as a function of junction area fraction (black solid dots) is well reproduced by the approximated analytical expression (black empty dots):2
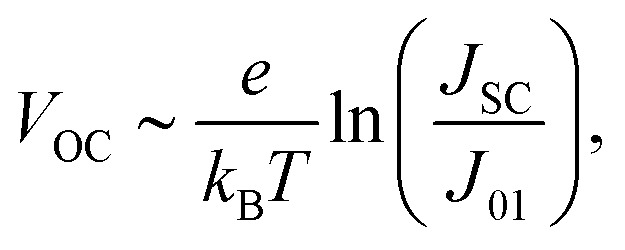
Here, *J*_01_ represents the reverse saturation current associated with recombination in the quasi-neutral regions and scales approximately linearly with the physical junction area (orange dots in Fig. 2b). This is because the number of available recombination sites in these regions increases proportionally with the contact area, leading to a proportional increase in the total reverse current. Thus, reducing the junction area fraction suppresses *J*_01_ and yields the observed voltage gains.

On the other hand, despite the gain in *V*_OC_, reducing the junction area also leads to a decrease in *J*_SC_ (Fig. 2c). Optical simulations show that absorption-related losses increase slightly with reducing the junction area, indicated by the empty dots in Fig. 2c. The increasing optical losses with reducing the junction area are due to two main reasons: (a) the absorber volume decreases, and (b) the additional SiO_2_ interface reduces the back-reflectance. However, these optical effects alone cannot fully account for the observed reduction in *J*_SC_. The relative difference *J*_ph_–*J*_SC_ (red-solid curve) increases non-linearly at small junction fractions, suggesting the presence of additional loss mechanisms. We attribute this behavior to current crowding and enhanced series resistance in the narrow openings, which limit carrier collection efficiency even when sufficient photogeneration occurs.

Fig. 2d shows the extracted series resistance *R*_series_, which rises exponentially as the hole radius shrinks. The current density distribution (inset) for the smallest hole radius of 30 nm (*i.e.* junction area fraction of 1.4%) confirms that carriers are funneled into confined pathways, increasing the resistance. These effects are consistent with previously reported models of spreading and access resistance.^33–35^ Therefore, we conclude that excessively small openings compromise current extraction and thus limit the total gain in efficiency. In this case, the optimal trade-off is achieved near 8% junction area fraction, where *V*_OC_ is enhanced without incurring current losses, leading to a modest PCE gain of less than 1%.

### 2.3 Effects of selective contact materials

Now, we explore the effect of contact material choice on the compromise between dark current and series resistance when reducing the junction area fraction. To do so, we maintain the Zn_3_P_2_ as the p-type absorber and vary the n-type carrier transport layer. In addition to InP, we consider Si and TiO_2_ (see Table S2 for the listed electrical characteristic values).

**Fig. 3:**
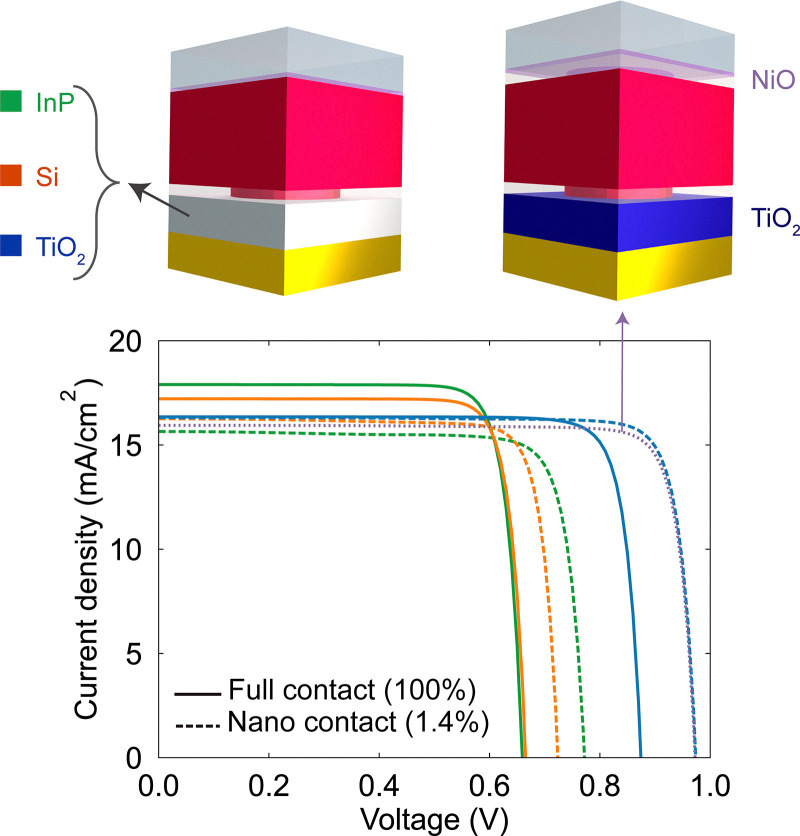
Simulated *JV* curves under 1 Sun illumination for different n-type emitters (InP-green, Si-orange, and TiO_2_-blue), at full contact (100%, solid curves) and with a nano-contact (1.4% contact area, dashed curves). We also show the simulated characteristics of a cell with nano-contacts at both the TiO_2_ emitter and at the hole selective layer NiO (purple dotted curve).

**Fig. 4:**
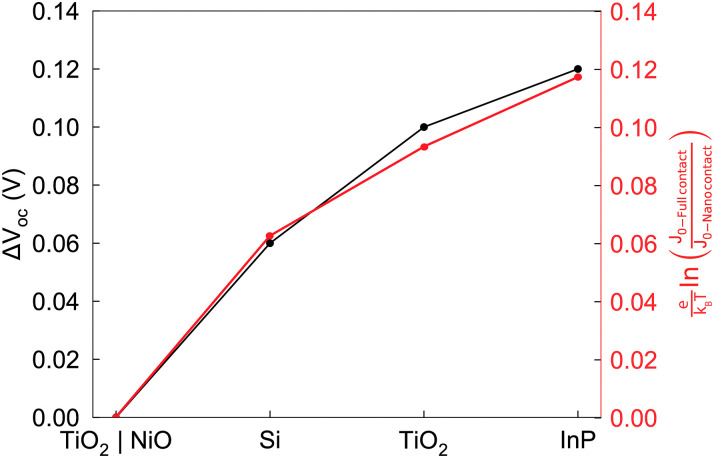
(Black) Relative increase in *V*_OC_ for the different contact materials explored in this work, from 100% to 1.4% of contact area fraction. (Red) Analytical approximation for the changes in open circuit voltage, considering only the contribution from *J*_01_.

Fig. 3 shows the simulated *JV* curves under illumination. We compare configurations with a full contact area (100%, solid curves) and a reduced junction area fraction of 1.4% (dashed curves), which we have previously found that maximizes *V*_OC_. The simulations confirm that the *V*_OC_ increases in all three cases when the junction contact area is reduced. The most significant improvements occur with InP and TiO_2_, with gains of 0.12 V and 0.10 V, respectively, while Si exhibits a more modest gain of 0.06 V (shown in Fig. 4, black dots). As expected from the previous section, changes in the *V*_OC_ are well justified by changes in the saturation current density, *J*_01_, as shown by the red dots in Fig. 4.

Similarly, the losses in *J*_SC_ are also material-dependent. The biggest loss is for InP, and it is almost negligible for TiO_2_. It is interesting to note that the degree of current loss follows the same trend as the absorptance of each material: InP (direct bandgap) generates significant carriers near the junction, Si (indirect bandgap) generates fewer, and TiO_2_ (wide bandgap) does not absorb (see Fig. S2a). We suspect that the contribution from an absorbing heterojunction partner is hampered by the larger access resistance at the nano-contacts, thereby reducing the total current. Thus, this effect is more prominent as the material is more absorbing.

Lastly, we also investigate whether reducing the contact area at the interface between Zn_3_P_2_ and a hole-selective contact (NiO) yields similar benefits. For this, we keep TiO_2_ as the n-contact layer. The simulated *JV* curve is shown as a purple dotted curve in Fig. 3. In this case, reducing the Zn_3_P_2_/NiO interface yields a negligible *V*_OC_ improvement, confirming that most recombination originates at the junction with the emitter. Therefore, geometric constraints at this front interface do not help further. Also, reducing the NiO/Zn_3_P_2_ interface introduces some optical losses due to the extra SiO_2_ layer with a non-optimized thickness for anti-reflectance, which slightly reduces *J*_SC_.

In terms of net efficiency, reducing the contact area with TiO_2_ offers the most favorable balance between voltage gain and current retention, achieving a PCE of up to 13.7%. This corresponds to an absolute increase of 1.45 percentage points (or a 12% relative improvement) attributable solely to the imposed geometric constraints.

## Conclusions

We present a geometric strategy to enhance the performance of thin-film solar cells by minimizing the p–n junction contact area, thereby reducing reverse saturation current and improving *V*_OC_ and PCE. The junction area fraction was tuned by patterning the n-type emitter (InP, Si, or TiO_2_) with a TiO_2_ layer and holes of different sizes, filled with Zn_3_P_2_. Reducing the contact area leads to a linear decrease in *J*_01_ and a corresponding increase in *V*_OC_; however, shrinking the hole radius also increases series resistance, resulting in current density losses. For InP, *V*_OC_ gains of up to 0.12 V are achieved for the smallest holes, while current losses rise exponentially, indicating the existence of an optimal junction size. Wide bandgap semiconductors like TiO_2_ are ideal for this method because they do not photo-generate carriers close to the junctions, avoiding an increase in *R*_series_ and current losses. Consequently, both the photocurrent contribution and recombination activity associated with the n-type contact become less significant, and the overall PCE is increasingly governed by the Zn_3_P_2_ absorber. For this reason, while the junction area fraction corresponding to maximum PCE may shift slightly depending on the chosen n-type material, the design conclusions regarding recombination suppression and performance trends remain general. Decreasing the p–n junction contact area is a promising strategy to enhance the solar cell's performance. It is evident from this study how voltage gain and current losses can be balanced by optimizing the junction size to the exact deflection point before the losses start to increase exponentially. This work provides a schematic guideline for optimizing stack design depending on material properties, *i.e.*, suitable n-type emitters and geometry, including junction contact area size.

Future research should look into the effects of interface recombination differences between the nanocontacts and the mask.

## Methods

Complete optoelectronic simulations of solar cell devices require both optical and electrical simulations.

### Optical simulations

Three-dimensional optical simulations were carried out using Ansys Lumerical software.^36^ Maxwell equations are solved with the finite-difference-time-domain (FDTD) method. Absorption and optical generation rate profiles are calculated within the active regions over the entire solar spectrum, normalized to the AM1.5 one. A plane wave light source with Bloch/periodic boundary conditions was employed covering the wavelength range of [300–1100] nm. The injection direction was normal to the surface of the solar cell. Due to the symmetry of the unit cell, Symmetry boundary conditions were used to reduce the simulation time and volume. Along the depth of the cell, instead, perfectly matched layer (PML) boundary conditions were used to prevent any reflections at the boundaries. An example of an optical simulation setup is shown in Fig. S6. The refractive index of the materials used was obtained from the literature (ITO,^37^ SiO_2_^38^ InP,^38^ Si,^38^ TiO_2_^39^ and Au^40^) or from ellipsometry measurements (NiO, Zn_3_P_2_).

### Electrical simulations

Three-dimensional electrical simulations were performed using Nextnano GmbH software.^41,42^ Poisson and Drift-diffusion equations are solved self-consistently with an iterative method. The optical generation profile, obtained from optical simulations, was integrated into the electronic tool. For the front and back contact, ohmic boundary conditions were applied to ensure flat-band alignment with the adjacent layers. The electrical parameters for the different materials are provided in Table S8 in SI. The radiative, Auger, and Shockley–Read–Hall recombination models were enabled. The non-linear Poisson equations were solved using a Newton solver with a residual tolerance of 1 × 10^−4^, as recommended for 3D simulations. The overall accuracy of the simulations was controlled by the residual parameter, varied between 1 × 10^−3^ and 1 × 10^−10^, which defines the convergence criterion as the difference between successive solutions of the differential equations within the iterative scheme. The *JV* curves were simulated by applying different voltages to the front contact in the range of [0, 1] V for the illuminated curve and [−1, 1] V for the case in the dark.

### Analysis

The data obtained from simulations have been analyzed with Python. In the graphs showing parameters trend as a function of the junction area fraction, *i.e.*, *V*_OC_ (Fig. S1a), *J*_SC_ (Fig. S1b), *FF*(Fig. S1c), PCE(Fig. 1c), *J*_01_ (Fig. S3b), *J*_02_ (Fig. S3c), *R*_series_ (Fig. S3d) and *R*_shunt_ (Fig. S3e), the data have been fitted with linear, exponential and two-exponential functions or interpolated with cubic-spline method. The details of the fits and functions used for each parameter can be found in the corresponding Figures in the SI.

## Author contributions

Melanie Micali: writing – original draft, investigation, conceptualization, methodology, formal analysis, conceptualization, visualization. Raphaël Lemerle: writing – review & editing, conceptualization. Anja Tiede: writing – review & editing, conceptualization. Anna Fontcuberta i Morral: writing – review & editing, conceptualization. Esther Alarcón Lladó: writing – review & editing, conceptualization, supervision.

## Conflicts of interest

The authors declare that they have no known competing financial interests or personal relationships that could have appeared to influence the work reported in this paper.

## Supplementary Material

YA-005-D5YA00251F-s001

## Data Availability

The data supporting this article have been included as part of the Supplementary information (SI). Supplementary information is available. See DOI: https://doi.org/10.1039/d5ya00251f.
