# Supplementary material for: Optimizing carrier collection in solar cells through nanoscale junction design
Source: Energy Adv. 2026 Mar 19;5(4):427–33. doi: 10.1039/d5ya00251f (PMC13001637; doi:10.1039/d5ya00251f)
Supplement: YA-005-D5YA00251F-s001 [file YA-005-D5YA00251F-s001.pdf]

Supplementary Information (SI) for Energy Advances.
This journal is © The Royal Society of Chemistry 2026

Supporting Information

S1 Effect of the InP/Zn3P2 junction area fraction

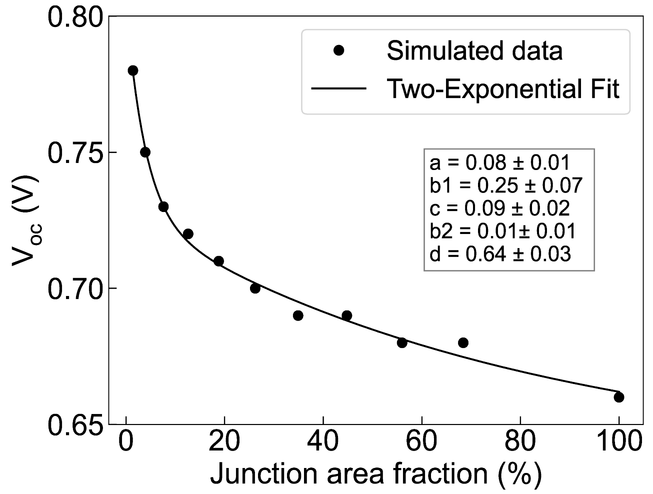

(a)

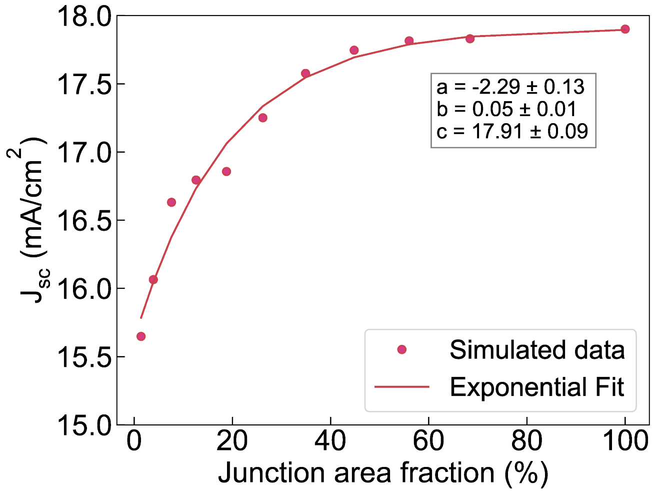

(b)

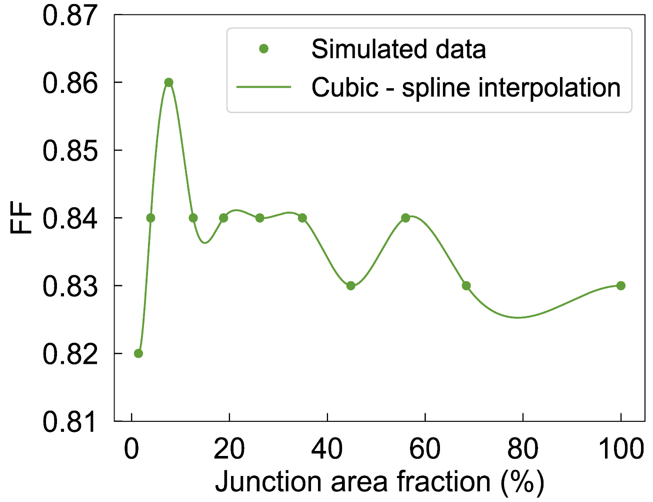

(c)

Figure S1 a) V_{oc} and b) J_{sc} trend as a function of the junction area fraction for the configuration featuring InP as the n-type emitter. The simulated data have been fitted with one-exponential ($y = a \cdot e^{-b \cdot x} + c$) and two-exponential($y = a \cdot e^{b1 \cdot x} + (c \cdot e^{b2 \cdot x} + d)$) functions, respectively. The fitting function, parameter fits, and standard deviations are reported in the legend and the insets. c) FF trend as a function of the junction area fraction for the configuration featuring InP as n-type emitter. The FF simulated data have been interpolated with a cubic-spline method.

Table S1 Junction area fraction, hole radius, V_{oc}, J_{sc}, FF, and PCE tabulated values for the configuration with InP as n-type emitter

Junction area fraction (%)	Hole radius (nm)	V_{oc} (V)	J_{sc} (mA/cm^2)	FF	PCE (%)
1.4	30	0.78	15.65	0.82	9.97
4	50	0.75	16.06	0.84	10.17
8	70	0.73	16.63	0.86	10.44
13	90	0.72	16.79	0.84	10.20
19	110	0.71	16.86	0.84	10.03
26	130	0.70	17.25	0.84	10.17
35	150	0.69	17.57	0.84	10.24
45	170	0.69	17.75	0.83	10.20
56	190	0.68	17.81	0.84	10.12
68	210	0.68	17.83	0.83	10.01
100	-	0.66	17.90	0.83	9.85

S2 Different n-type emitters

Table S2 Interfaces with reduced contact area, junction area fraction, V_{oc}, J_{sc}, FF and PCE tabulated values for the different structures

Interfaces with reduced contact area	Junction area fraction (%)	V_{oc}(V)	J_{sc}(mA/cm^2)	FF	PCE(%)
InP/Zn_3P_2	100	0.66	17.90	0.83	9.85
	1.4	0.78	15.65	0.82	9.97
Si/Zn_3P_2	100	0.67	17.22	0.83	9.61
	1.4	0.73	16.26	0.83	9.80
TiO_2/Zn_3P_2	100	0.88	16.35	0.85	12.22
	1.4	0.98	16.31	0.86	13.67
TiO_2/Zn_3P_2 and Zn_3P_2/NiO	1.4 - 1.4	0.98	15.94	0.86	13.34

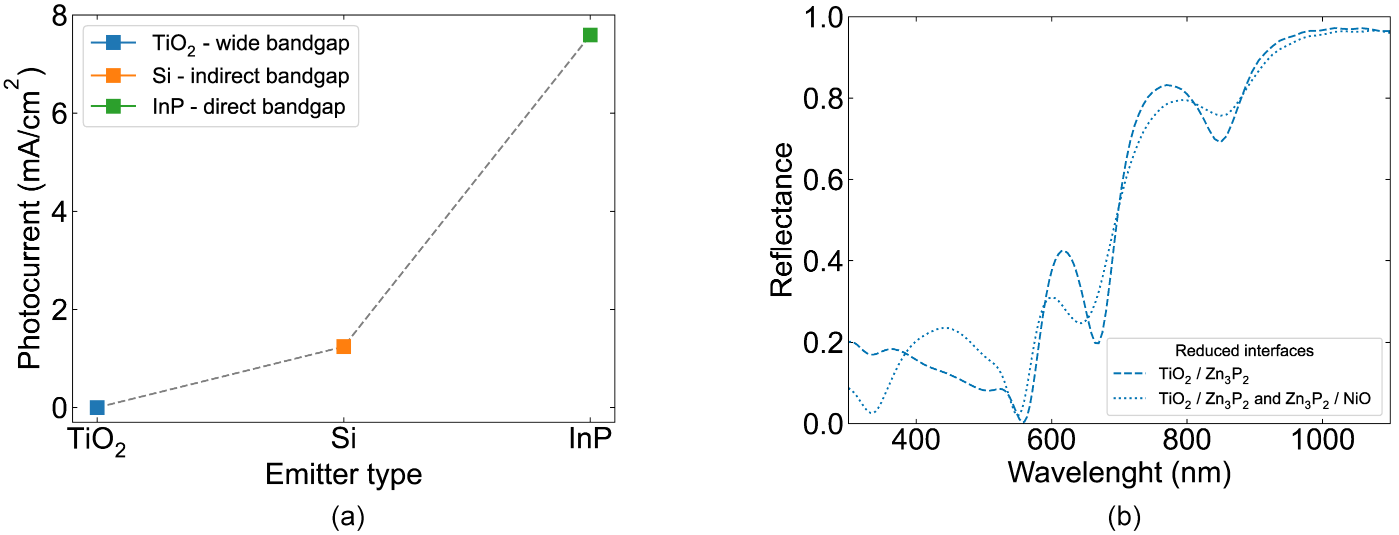

Figure S2 a) J_{ph} contributions from the different n-type emitters for a junction area fraction $= 100\%$. b) Reflectance comparison for the structures where one interface (TiO_2/Zn_3P_2) is reduced at 1.4% and two interfaces (TiO_2/Zn_3P_2 and Zn_3P_2/NiO) are reduced at 1.4% and 1.4%.

S3 2Diode and 3Diode models for the fit of the dark JV curves

The current-voltage characteristics of a solar cell in the dark can be expressed by the non-ideal diode form[1]:

$$\mathrm{J(V)} = J_{sc} - J_{n,0}(e^{\frac{qV}{nkbT}} - 1) \qquad \text{(eq.S1)}$$

where $J_{n,0}$ is the saturation current in the dark, J_{sc} the short circuit current, k_B and T the Boltzmann constant and the temperature of the solar cell, respectively. The ideality factor n expresses the dominant type of dark current when bulk, junctions, and surface recombination have different bias dependencies. The expression for V_{oc} can be found for $J = 0$ in eq.S1:

$$V_{oc} = \frac{nk_BT}{q} \ln\left(\frac{J_{sc}}{J_{n,0}} + 1\right) \qquad \text{(eq.S2)}$$

The dark current exhibits $n = 2$ at low positive bias, indicative of the recombination at the junction. At higher forward bias, when recombination in the bulk regions dominates, it is found $n = 1$. A single diode equation (eq.S1) assumes a constant value for n. However, in a real solar cell n can vary as a function of the voltage. A double-diode model can then be implemented to include the voltage dependence and the different predominant recombination mechanisms. The current-voltage expression assumes the form:

$$J = J_{\mathrm{ph}} - J_{01}\left(e^{\frac{q(V-JR_{\mathrm{series}})}{kT}} - 1\right) - J_{02}\left(e^{\frac{q(V-JR_{\mathrm{series}})}{2kT}} - 1\right) - \frac{V - JR_{\mathrm{series}}}{R_{\mathrm{shunt}}} \qquad \text{(eq.S3)}$$

A second diode is added in parallel with the first. For both diodes, a saturation current can be extracted, J_{01} and J_{02}, at higher and lower voltages, respectively. Series resistances R_{series} and Shunt resistances R_{shunt} are included in the model and can also be calculated from the above equation. The different dark JV curves have been simulated for the different junction area fractions in a voltage range $V = [-1, 1]V$ as shown in Figure S3a and for the configurations with different n-type emitters (Figure S4a, S4b, S4c, and S4d).
The curves for the Si and TiO_2 case show a characteristic broad shoulder at low-to-moderate voltages in the range $V = [0.2, 0.4]V$. This behavior is not consistent with the 2-diode equivalent circuit, as reported by McIntosh et al.[2,3]. The circuit should be modified with the addition of a third diode and an additional resistor. Several mechanisms can lead to an $n - V$ hump: floating-junction shunting, localized small regions in the solar cell with high recombination rate restively isolated from the main body of the cell, and field-enhanced depletion-region recombination. It is however, reported, that it is also possible that the hump can be caused by any of these mechanisms[2].
We used the *2/3-Diode Fit* software[4] to fit the curves. Depending on the presence of the characteristic $n - V$ hump, 2-diode or 3-diode models have been implemented. Tables S4, S5, S6, and S7 contain the diode-model used to fit the data, and the corresponding parameters extracted from the fits for the different simulation sets. In Figure S3, the parameters extracted from the fits are plotted as a function of the junction area fractions for the set of simulations where InP is used as n-type emitter.

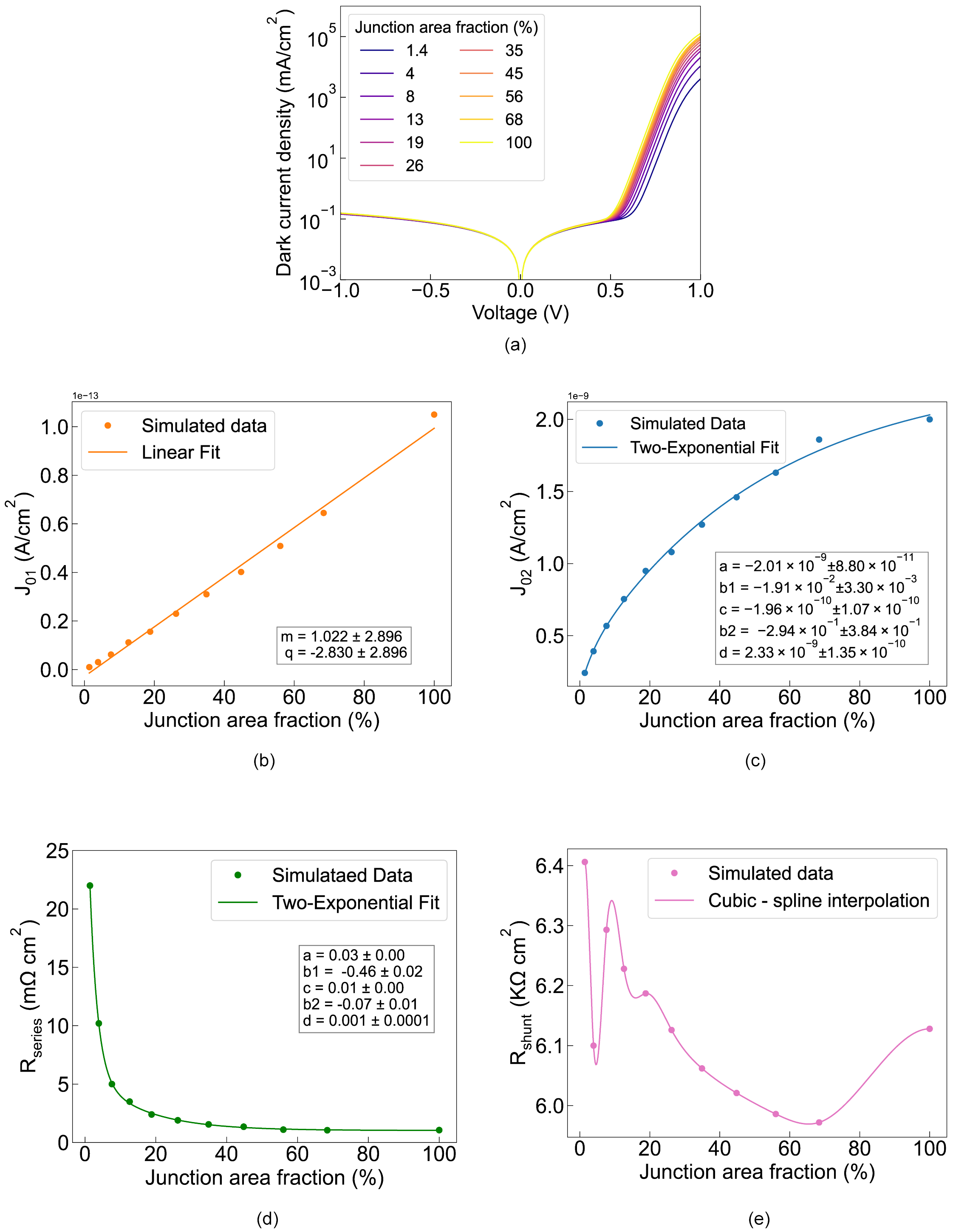

Figure S3 a) Simulated dark JV curves for different InP/Zn_3P_2 junction area fractions. Parameters trend as a function of the junction area fractions extracted from the fit, with a 2-diode model, of the dark JV curves: b) J_{01}, c) J_{02}, d) R_{series}, e) R_{shunt}. The data have been fitted with a linear ($y = m \cdot x + q$) and two-exponential($y = a \cdot e^{b1 \cdot x} + (c \cdot e^{b2 \cdot x} + d)$) functions, or interpolated with a cubic-spline method. The fitting function, parameter fits, and standard deviations are reported in the legend and in the insets.

Table S3 Tabulated values of the parameters extracted from the fit of the dark JV curves with the 2-diode model when InP is the n-type emitter material: Junction area fraction, J_{01}, J_{02}, R_{series}, and R_{shunt}

Junction area fraction (%)	J_{01} (10^{-14}A/cm^2)	J_{02} (10^{-9}A/cm^2)	R_{series} ($10^{-3}\Omega$cm^2)	R_{shunt} ($10^{3}\Omega$cm^2)
1.4	0.1	0.2	22.0	6.41
4	0.3	0.4	10.2	6.36
8	0.6	0.6	5.00	6.29
13	1.1	0.7	2.80	6.23
19	1.6	0.9	2.40	6.19
26	2.3	1.1	1.90	6.13
35	3.1	1.3	1.55	6.06
45	4.0	1.5	1.30	6.02
56	5.1	1.6	1.10	5.98
68	6.5	1.9	1.05	5.97
100	11.0	2.0	1.06	6.14

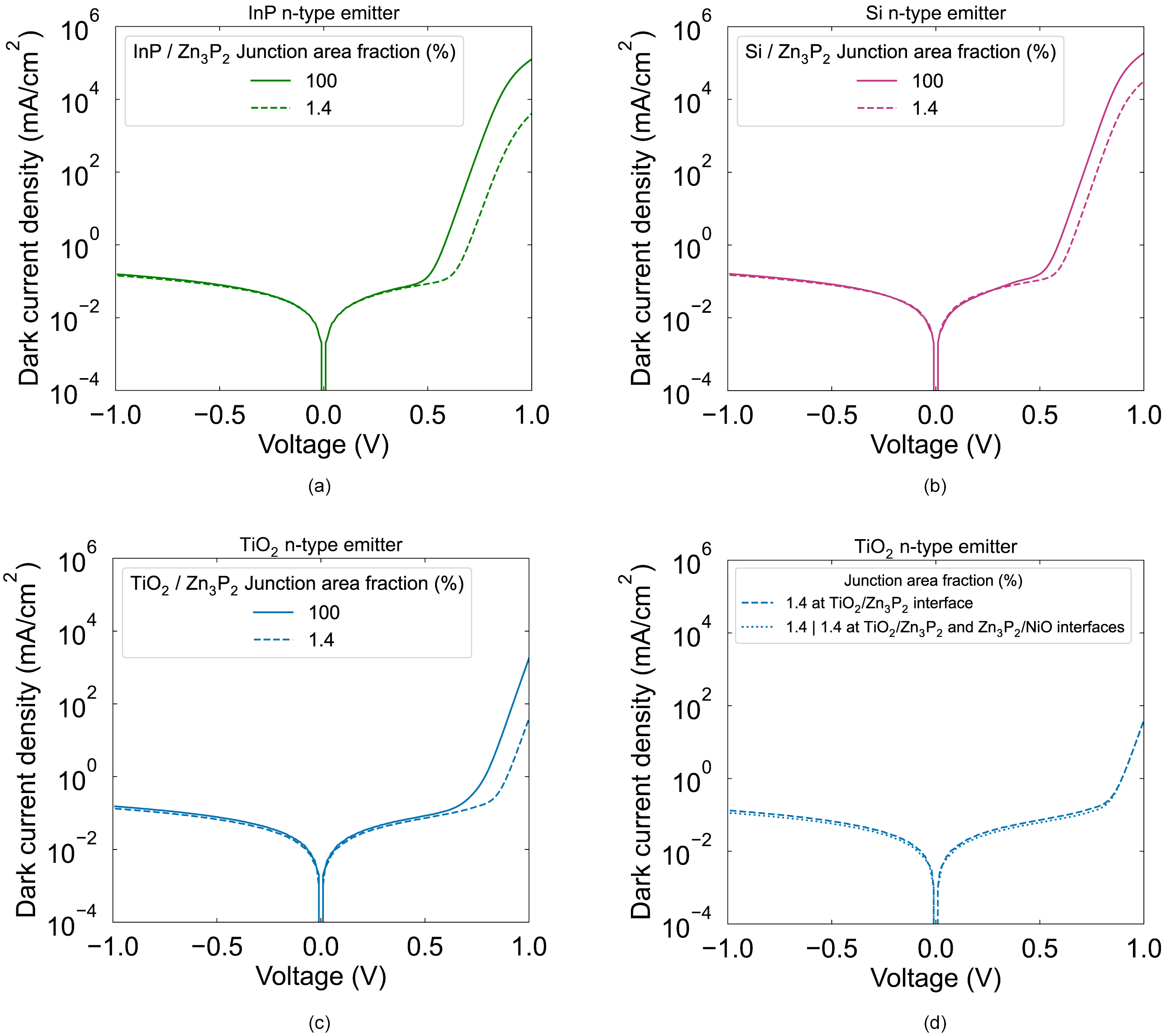

Figure S4 Comparison of the simulated dark JV curves with a junction area fraction of 100% and 1.4% for the different interfaces reduced: a) InP/Zn_3P_2, b) Si/Zn_3P_2, c) TiO_2/Zn_3P_2, and d) TiO_2/Zn_3P_2 with Zn_3P_2/NiO.

Table S4 Parameters extracted from the fit of the dark JV curves with the 2-diode model when InP is the n-type emitter material: Junction area fraction, J_{01}, J_{02}, R_{series}, and R_{shunt}

Junction area fraction (%)	J_{01} (10^{-14}A/cm^2)	J_{02} (10^{-9}A/cm^2)	R_{series} ($10^{-3}\Omega$cm^2)	R_{shunt} ($10^3\Omega$cm^2)
100	11.0	2.0	2.06	6.14
1.4	0.1	0.2	22.0	6.41

Table S5 Parameters extracted from the fit of the dark JV curves with the 3-diode model when Si is the n-type emitter material: Junction area fraction, J_{01}, J_{02}, J_{03}, R_{series}, R_{shunt}, and R_h

Junction area fraction (%)	J_{01} (10^{-14}A/cm^2)	J_{02} (10^{-10}A/cm^2)	J_{03} (10^{-6}A/cm^2)	R_{series} ($10^{-3}\Omega$cm^2)	R_{shunt} ($10^3\Omega$cm^2)	R_h ($10^3\Omega$cm^2)
100	9.9	4.1	1.1	0.5	6.1	4.4
1.4	0.8	0.9	0.8	2.9	7.0	9.8

Table S6 Parameters extracted from the fit of the dark JV curves with the 3-diode model when TiO_2 is the n-type emitter material: Junction area fraction, J_{01}, J_{02}, J_{03}, R_{series}, R_{shunt}, and R_h

Junction area fraction (%)	J_{01} (10^{-14}A/cm^2)	J_{02} (10^{-10}A/cm^2)	J_{03} (10^{-6}A/cm^2)	R_{series} ($10^{-3}\Omega$cm^2)	R_{shunt} ($10^3\Omega$cm^2)	R_h ($10^3\Omega$cm^2)
100	2.1	1.0	1.1	5.7	6.3	4.4
1.4	0.05	0.04	-	5.7	7.5	-

Table S7 Parameters extracted from the fit of the dark JV curves with 2-diode and 3-diode models when TiO_2 is the n-type emitter material and one or two Zn_3P_2 interfaces are reduced: Junction area fraction, J_{01}, J_{02}, J_{03}, R_{series}, R_{shunt}, and R_h.

Junction area fraction (%)	J_{01} (10^{-14}A/cm^2)	J_{02} (10^{-10}A/cm^2)	J_{03} (10^{-6}A/cm^2)	R_{series} ($10^{-3}\Omega$cm^2)	R_{shunt} ($10^3\Omega$cm^2)	R_h ($10^3\Omega$cm^2)
1.4	0.05	0.04	1.1	5.7	7.5	3.5
1.4 | 1.4	0.05	0.04	1.1	5.7	8.7	7.0

S4 Optical simulation set-up

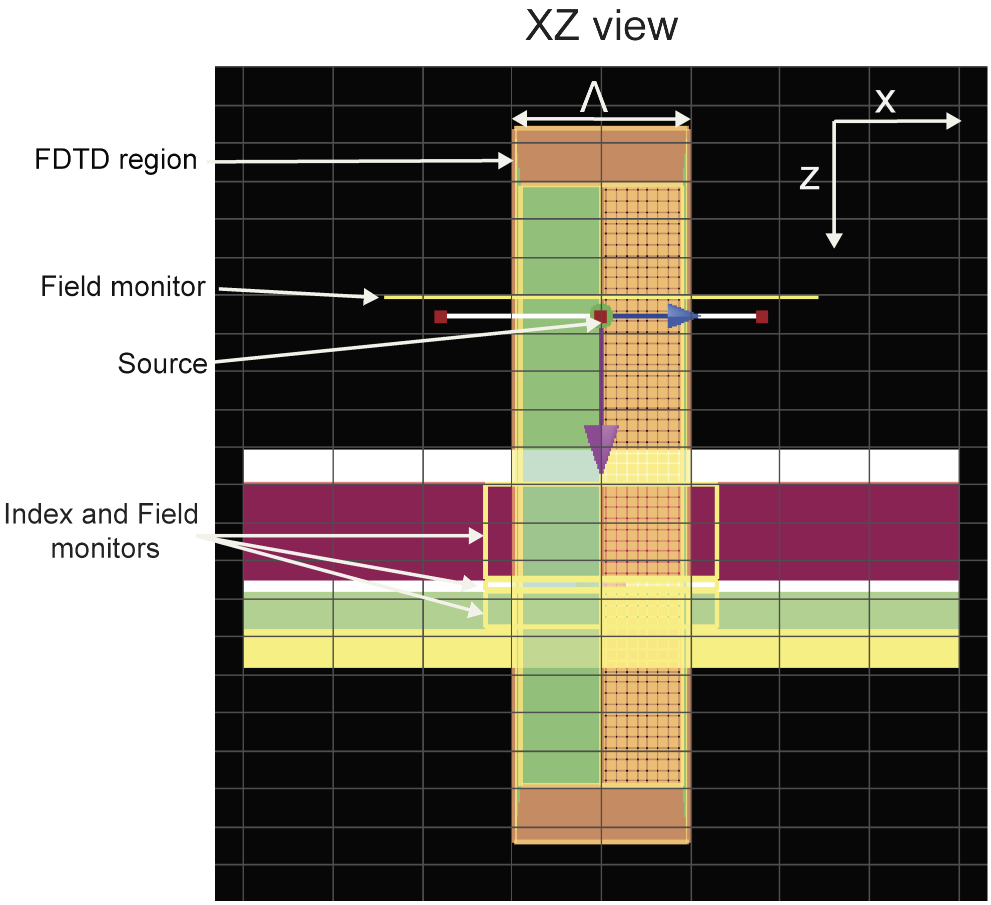

Figure S5 Optical simulation set-up used in FDTD Ansys Lumerical software.

S5 Material parameters

Table S8 List of semiconductor parameters for the Poisson and Drift-diffusion simulations

Property	Units	Zn_3P_2	NiO	SiO_2	InP [5,6]	Si [5,6]	TiO_2
Thickness	nm	250	10	30	100	100	100
DC permittivity	-	11 [?,7]	10.7	3.9	12.61	11.9	66
Valence band offset	eV	-5.11 [8]	-5.26	-9.8	-5.72	-5.16	-7.2
E_c valley	-	Γ	Γ	X	Γ	X	Γ
Effective mass electrons	$1/m_e$	0.268 [9]	1.07	0.98	0.0795	0.98	0.24
Effective mass holes	$1/m_e$	1.055 [9]	0.54	0.537	0.6	0.537	0.8
Bandgap	eV	1.51 [10]	3.8	9.0	1.35	1.12	3.2
Mobility electrons	cm^2/Vs	1000 [11]	12	142	1000	105.9	20
Mobility holes	cm^2/Vs	20 [12]	2.8	47	30	68.7	5
N-type doping	cm^{-3}	-	-	-	$1 \cdot 10^{19}$	$1 \cdot 10^{19}$	$1 \cdot 10^{19}$
P-type doping	cm^{-3}	$1 \cdot 10^{16}$	$1 \cdot 10^{19}$	-	-	-	-
Electron zero-doping scattering time (SRH mechanism)	s	$4.6 \cdot 10^{-9}$ [13]	$4.26 \cdot 10^{-4}$	$4.26 \cdot 10^{-4}$	$1 \cdot 10^{-8}$	$4.26 \cdot 10^{-4}$	$1.0 \cdot 10^{-9}$
Hole zero-doping scattering time (SRH mechanism)	s	$4.6 \cdot 10^{-9}$ [13]	$3.95 \cdot 10^{-4}$	$3.95 \cdot 10^{-4}$	$3 \cdot 10^{-6}$	$3.95 \cdot 10^{-4}$	$1.0 \cdot 10^{-9}$
Auger electron capture coefficient	cm^6/s	$3.70 \cdot 10^{-31}$	$2.1 \cdot 10^{-31}$	$2.8 \cdot 10^{-31}$	$1.7 \cdot 10^{-33}$	$2.8 \cdot 10^{-31}$	$1.7 \cdot 10^{-33}$
Auger hole capture coefficient	cm^6/s	$8.70 \cdot 10^{-30}$	$2.1 \cdot 10^{-31}$	$9.9 \cdot 10^{-31}$	$9 \cdot 10^{-31}$	$9.9 \cdot 10^{-32}$	$9 \cdot 10^{-31}$
Radiative recombination coefficient	cm^3/s	$1.6 \cdot 10^{-11}$	$2.3 \cdot 10^{-9}$	$1.1 \cdot 10^{-14}$	$2 \cdot 10^{-10}$	$4.73 \cdot 10^{-15}$	$2 \cdot 10^{-10}$

References

[1] J. A. Nelson, *The physics of solar cells*, World Scientific Publishing Company, 2003.

[2] K. McIntosh, *PhD thesis*, UNSW Sydney, 2001.

[3] F. Hernando, R. Gutierrez, G. Bueno, F. Recart and V. Rodriguez, Proc, 1998, p. 1321.

[4] S. Suckow, *URL: https://nanohub. org/resources/14300*, 2014.

[5] O. Madelung, *Semiconductors: data handbook*, Springer Science & Business Media, 2004.

[6] A. Dargys and J. Kundrotas, *Vilnius: Science and Encyclopedia Publishers, 1994.*, 1994.

[7] J. Misiewicz, J. Wrobel and B. Clayman, *Solid state communications*, 1988, **66**, 747–750.

[8] J. P. Bosco, D. O. Scanlon, G. W. Watson, N. S. Lewis and H. A. Atwater, *Journal of Applied Physics*, 2013, **113**, 203705.

[9] J. P. Bosco, *Rational design of zinc phosphide heterojunction photovoltaics*, California Institute of Technology, 2014.

[10] M. Dimitrievska, F. S. Hage, S. Escobar Steinvall, A. P. Litvinchuk, E. Z. Stutz, Q. M. Ramasse and A. Fontcuberta i Morral, *Advanced Functional Materials*, 2021, **31**, 2105426.

[11] G. M. Kimball, A. M. Müller, N. S. Lewis and H. A. Atwater, *Applied Physics Letters*, 2009, **95**, 112103.

[12] J. Pawlikowski, *Optica Applicata*, 1989, **19**, 31–50.

[13] E. Z. Stutz, M. Zamani, D. A. Damry, L. Buswell, R. Paul, S. E. Steinvall, J.-B. Leran, J. L. Boland, M. Dimitrievska and A. F. i Morral, *Materials Advances*, 2022, **3**, 1295–1303.
